# P36 Procalcitonin as an antimicrobial stewardship tool in the COVID-19 era: a single centre experience

**DOI:** 10.1093/jacamr/dlac004.035

**Published:** 2022-02-16

**Authors:** Margaret Creedon, Alida Fe Talento, Roisin Connolly, Aine Egan, Tidi Hassan, Marta Trzos-Grzybowksa

**Affiliations:** Our Lady of Lourdes Hospital, Drogheda, Ireland; Our Lady of Lourdes Hospital, Drogheda, Ireland; Our Lady of Lourdes Hospital, Drogheda, Ireland; Our Lady of Lourdes Hospital, Drogheda, Ireland; Our Lady of Lourdes Hospital, Drogheda, Ireland; Our Lady of Lourdes Hospital, Drogheda, Ireland

## Abstract

**Background:**

Procalcitonin (PCT) is a biomarker which may have greater specificity than other pro-inflammatory markers in identifying bacterial infection and may aid in antimicrobial stewardship in COVID-19 patients.

**Methods:**

A PCT assay by Abbott diagnostics was introduced in our hospital in November 2020. Over a 12 week period, we prospectively recorded all PCT results on in-patients with COVID-19 admitted to the ICU, as well as stewardship actions taken following discussion with the ICU physicians. Microbiology records were reviewed to ascertain whether bacterial infection was subsequently confirmed.

**Results:**

Sixty-four PCT results were recorded on 27 patients (Table 1). Antimicrobials were discontinued on 12 occasions and withheld on 16 occasions where PCT was <0.5 ng/mL. No cases of confirmed bacterial infection were diagnosed in patients with PCT concentrations of <0.05 ng/mL; however definite bacterial infection was confirmed in two patients (one case of *Staphylococcus aureus* bloodstream infection; one case of *Staphylococcus epidermidis* CVC infection), and bacterial infection was designated probable in five patients with PCT ≥0.05 ng/mL and <0.5 ng/mL.

**Conclusions:**

PCT may be a useful tool in enabling antimicrobial stewardship actions in patients with severe COVID-19, but results need to be interpreted within the clinical context.

**Table 1.** PCT results

Result	Interpretation	No. of samples
<0.05 ng/mL	Absence of bacterial infection	4
≥0.05 ng/mL and <0.5 ng/mL	Systemic bacterial infection is not likely	41
≥0.5 ng/mL and <2 ng/mL	Systemic bacterial infection is possible	12
≥2 ng/mL and <10 ng/mL	Systemic bacterial infection is likely	5
≥10 ng/mL	Systemic bacterial is highly likely	2

